# Very Long Term Stability of Mixed Chimerism after Allogeneic Hematopoietic Stem Cell Transplantation in Patients with Hematologic Malignancies

**DOI:** 10.1155/2015/176526

**Published:** 2015-11-10

**Authors:** Emmanuel Levrat, Eddy Roosnek, Stavroula Masouridi, Bilal Mohty, Marc Ansari, Jean Villard, Jakob R. Passweg, Yves Chalandon

**Affiliations:** ^1^Department of Hemato-Oncology, Hôpital Fribourgeois, 1708 Fribourg, Switzerland; ^2^Division of Hematology, Geneva University Hospital, 1205 Geneva, Switzerland; ^3^Transplantation and Cellular Therapy Unit, Paoli-Calmettes Institute, 13009 Marseille, France; ^4^Division of Hematology, Basel University Hospital, 4056 Basel, Switzerland

## Abstract

The objective of this study is to analyze the evolution of chimerism of all patients transplanted for hematologic malignancies in our unit during a 20-year period, alive without relapse at 1 year after allogeneic hematopoietic stem cell transplantation (HSCT). Chimerism was tested using short tandem repeat polymorphisms after separation into mononuclear cells and granulocytes by Ficoll density gradient centrifugation. Of 155 patients studied, 89 had full chimerism (FC), 36 mononuclear cells mixed chimerism (MNC-MC), and 30 granulocytic MC with or without mononuclear cells MC (Gran-MC). Survival was significantly better in MNC-MC than in Gran-MC patients, with FC patients being intermediate. There was more disease relapse in the Gran-MC group but not in the MNC-MC group as compared to FC. MC was stable up to 21 years in the MNC-MC group and up to 19 years in the Gran-MC group. Of MC patients alive at 10 years, MC persisted in 83% in the MNC-MC and 57% in the Gran-MC groups. In conclusion, mixed chimerism may remain stable over a very long time period. In survivors without relapse at 1 year after HSCT, determining lineage specific chimerism may be useful as outcome differs, MNC-MC being associated with better outcome than Gran-MC.

## 1. Introduction

Allogeneic hematopoietic stem cell transplantation (HSCT) is usually undertaken to replace the recipient by donor hematopoiesis resulting in full donor chimerism (FC), a state where alleles detected in the blood of the patient after HSCT are of donor origin [1]. HSCT may result, however, in states of mixed hematopoietic chimerism (MC), especially after reduced intensity conditioning regimens [2] and after T-cell depletion [3]. MC can have multiple meanings and clinical implications, may occur in different cellular compartments, and may have a varying course over time. Increasing MC levels in HSCT performed after hematological malignancies may indicate disease relapse, graft failure, or rejection. On the other hand, decreasing MC, often seen after tapering of immunosuppression after transplant or after donor lymphocytes infusion (DLI), may be an early predictor of graft-versus-host disease (GvHD) and of its more desirable counterpart graft-versus-tumor effect, although the correlation between these 2 entities is not very close. Furthermore, MC may remain stable over time and be compatible with prolonged remission, particularly in nonmalignant diseases, where MC may indicate a tolerant state associated with a low incidence of GvHD [4, 5]. Eventually, determining chimerism may also be useful to monitor response to a DLI or help to decide on administering prophylactic DLI in specific situations (e.g., to potentiate graft-versus-tumor effect [6–8] or to prevent incipient graft rejection [9] in some cases of increasing MC).

The goals of this observational study are to analyze the stability and evolution of MC in allogeneic HSCT patients with hematologic malignancies, alive without relapse at 1 year after HSCT, and to compare the outcomes of patients with “lineage restricted” MC (granulocytes versus mononuclear cells chimerism), as performed in our institution, with those of patients with FC. While granulocytic MC recipient myelopoiesis is considered to be contributing to the total myelopoiesis in a given patient at the time point of analysis, mononuclear MC is used to define lymphoid MC.

## 2. Patients and Methods

We have retrospectively analyzed data of all patients receiving an allogeneic HSCT in our institution between 1986 and 2006 for hematologic malignancies, alive without relapse at 1 year after HSCT. This inclusion criterion was selected to eliminate early posttransplant MC reflecting early relapse. Furthermore, some reports have already demonstrated the role of posttransplant MC at 3 months [10] or at 6 months [11] after HSCT as a predictor of relapse in hematopoietic malignancies.

T-cell depletion was done in all patients with low risk disease defined as first complete remission (CR1), or first chronic phase (CP1) for chronic myeloid leukemia, and for most patients with intermediate and advanced risk disease but not for patients with active leukemia at the time of transplantation. T-cell depletion was performed with CAMPATH-1M in the early years, followed with CAMPATH-1G and finally with CAMPATH-1H in the bag as described by Chalandon et al. [12]. Protocols varied and included T-cell depletion without T-cell add-back in the early years (before 1998) and later T-cell depletion with T-cell add-back (after 1998).

Chimerism was determined on peripheral blood, using short tandem repeats (STR) polymorphisms on informative loci, as described elsewhere [13, 14], after separation, by Ficoll density gradient centrifugation, into granulocytes (Gran) and mononuclear cells (MNC), reflecting lymphocytes and monocytes. After the first year after transplant, chimerism testing was performed on peripheral blood at least annually in all patients, sometimes more frequently in those with persistent MC. MC level of detection was approximately 3% of recipient alleles by planimetric measurement.

All statistical analyses were performed with SPSS Statistics 13.0 software (SPSS, Chicago, IL, USA). The significance level was 0.05. Groups were compared using nonparametric tests for continuous and the chi-squared statistic for categorical variables. Survival analysis was by the Kaplan-Meier estimator and comparisons among groups were by the log-rank test and cumulative incidence was used for relapse with death without relapse defined as the competing risk.

## 3. Results

Of 259 patients having an allogeneic HSCT during the study period, 155 patients (60%) fulfilled the inclusion criteria. Of these patients, 89 patients had FC, 36 mononuclear cells MC (MNC-MC), and 30 granulocytic MC with or without mononuclear cells MC (Gran-MC). Ninety-three were males (60%) and 62 were females (40%). Median age was 38 years (range, 5–66 years). Median follow-up was 8.8 years (range, 1.1–21 years).  Table 1 shows the distribution of chimerism status based on main transplant features. There were no differences regarding to sex, disease, conditioning, donor type, stem cell source, and donor lymphocyte infusions between the 3 groups (*p* > 0.05), but there was more MC in patients with T-cell depletion (*p* < 0.001) and less MC in patients with acute graft-versus-host disease (GvHD) (*p* = 0.045), chronic GvHD (*p* = 0.009), and female donor into male recipient HSCT (*p* = 0.001).

**Figure 1:**
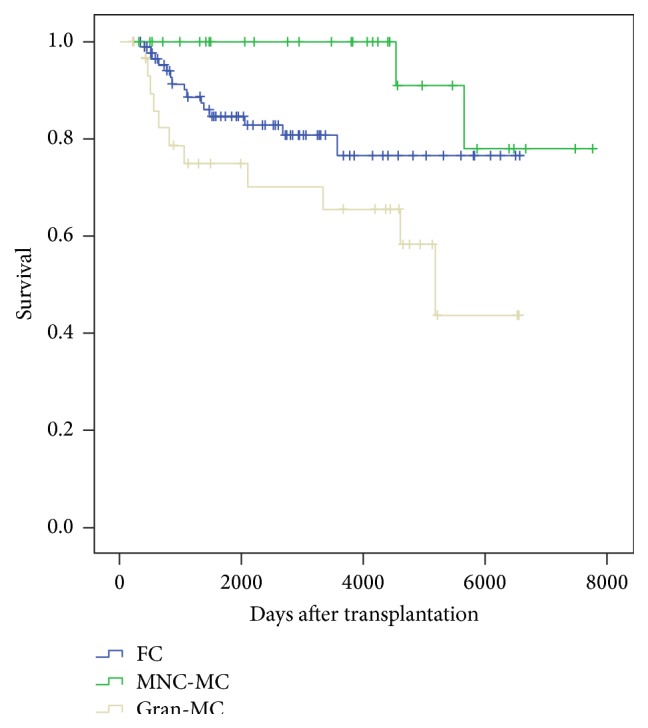
Overall survival according to chimerism status.

Figure 1 shows the overall survival (OS) of allogeneic HSCT, according to their chimerism status. Among these patients, OS was significantly better in MNC-MC group than in Gran-MC group (*p* = 0.001), with FC patients being intermediate.

**Figure 2:**
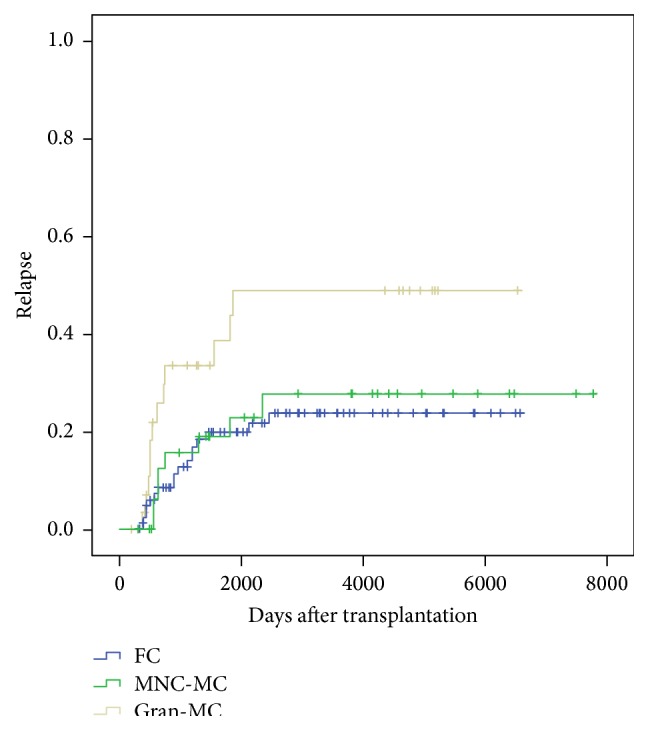
Relapse incidence according to chimerism status.

Figure 2 shows the cumulative incidence of relapse of allogeneic HSCT patients, according to their chimerism status. There was more disease relapse in the Gran-MC group but not in the MNC-MC group as compared to FC (*p* = 0.026).

**Figure 3:**
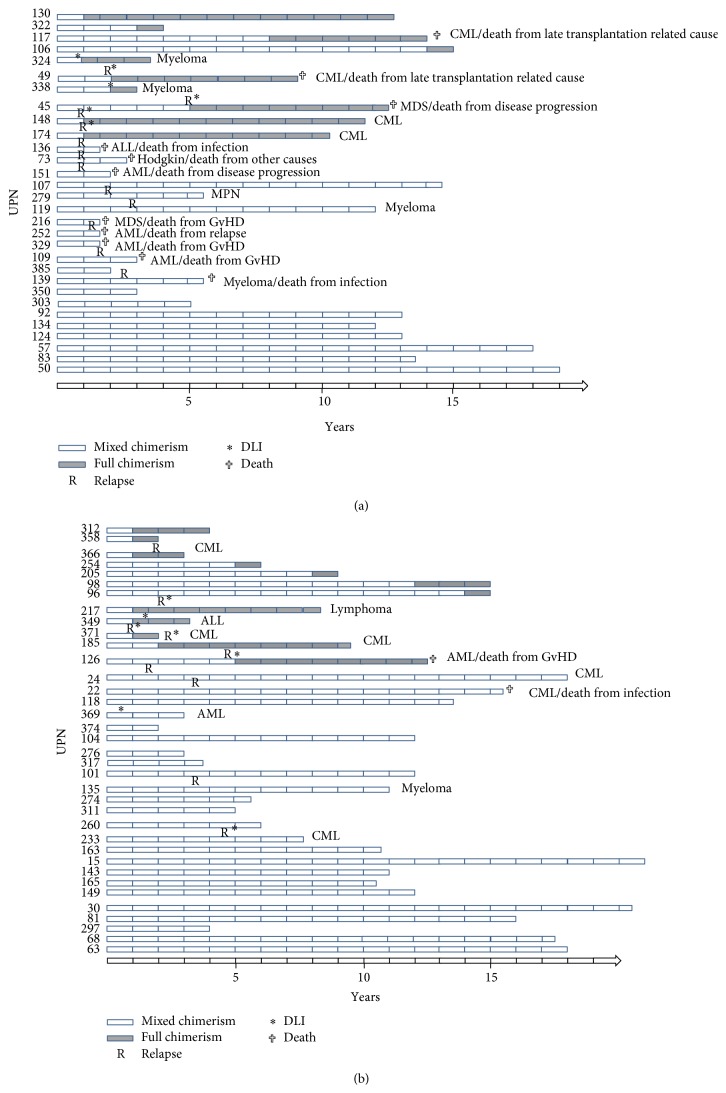
(a) Chimerism evolution in patients with Gran-MC. (b) Chimerism evolution in patients with MNC-MC.

MC was stable over prolonged periods in some patients in the MNC-MC and the Gran-MC groups (up to 21 and 19 years, resp.). Among MC patients alive at 10 years, MC persisted in 57% (8/14) in the Gran-MC group and 83% (15/18) in the MNC-MC group (*p* = 0.10; see Figures 3(a) and 3(b) for graphical display of the duration of MC stability). 10/30 patients in the Gran-MC group lost MC because of transformation to FC, 4 patients lost MC spontaneously, and 6 patients lost MC following DLI (4 DLI administrated because of relapse). 12/36 patients in the MNC-MC group lost MC because of transformation to FC, 7 patients lost MC spontaneously, and 5 patients lost MC following DLI (4 DLI administrated because of relapse).

## 4. Discussion

Our study shows that MC is compatible with prolonged survival in HSCT recipients, even in patients with hematologic malignancies, the longest follow-up being more than 20 years. Although MC in some patients converted either spontaneously or after DLI into FC, in others, MC remained stable during a very long time, with 72% (23/32) of persisting MC among all MC patients alive at 10 years. This represents a cohort with hematological malignancies with long follow-up with persisting MC. Persisting MC, defined as MC for more than 2 years in patients without evidence of relapse, was already reported in 19 patients with hematological malignancies, with a median leukemia free survival of 12.5 (range, 4.1–18.1) years [15]. Furthermore, long term stable MC has also been reported in patients with nonmalignant diseases, with MC in 12 patients over a median period of 9.5 (range, 5–16.5) years after HSCT [16]. In that study, despite limited patient cohort, a multivariate analysis showed that sibling donor was associated with stable MC. Additionally, development of acute GvHD and blood stream infection was significantly more prevalent in the FC patient group.

There are several limitations to our study. It is retrospective and limited to patients with hematological malignancies. Furthermore, chimerism measurements were made on peripheral blood samples, which can restrict the sensitivity of MC detection compared to bone marrow determination [17]. Finally, the sensitivity of the MC detection level (approximately 3%) of our method and its operator-dependent aspect, with potential contamination risk of the mononuclear cells by granulocytes contained in the Ficoll gradient during processing, should lead to careful interpretation.

Different researchers have used a variety of techniques for detection of chimerism, each with its merits and its pitfalls [18]: erythrocyte phenotyping, cytogenetic analysis, fluorescent in situ hybridization, restriction fragment length polymorphism, short tandem repeats (STR)/variable number tandem repeats (VNTR) analysis, X- or Y-chromosome markers, and amelogenin. Some groups have developed more sensitive techniques for quantitative evaluation of mixed chimerism using real-time polymerase chain reaction (PCR), like real-time single nucleotide polymorphism-PCR [19], potentially superior to standard tandem repeats-PCR for detection of MC, or sequence polymorphism based-PCR [20] which provide a rapid and accurate evaluation of MC. However, techniques based on STR/VNTR like our in-house method, although less sensitive, still remain very informative and useful for detection and quantitative assessment of MC.

Our patients with mixed chimerism are more likely to have received transplants T-cell depleted by CAMPATH (48% (65/135) of MC after CAMPATH T-cell depletion versus 5% (1/20) of MC without CAMPATH T-cell depletion). Patients with FC are more likely to have developed significant acute GvHD (grades 2 to 4) or chronic GvHD: 74% (29/39) of patients with significant acute GvHD and 79% (37/47) of patients with chronic GvHD had FC. Interestingly, similar high incidence of MC with low risk of acute and chronic GvHD has also been demonstrated in patients receiving alemtuzumab containing conditioning, but without increased relapse risk in MC patients [21, 22], contrary to our Gran-MC group (Figure 2) whose relapse risk was greater than in the FC group.

In relapse-free survivors at 1 year after HSCT, determining “lineage restricted” chimerism using a simple Ficoll density gradient centrifugation based method of separation by granulocytic and mononuclear cell chimerism may be useful as outcome differs, MNC-MC being associated with better outcome than Gran-MC. MNC-MC probably reflects the persistence of T lymphocytes/T-cell lymphopoiesis that have survived the conditioning, a situation not associated with increased relapse risk. Miura and colleagues already reported that donor-type chimerism in lineage-specific cell populations (CD3+, CD14.15+, and CD56+ cells in their study) appears to have an impact on outcome after HSCT [23].

## 5. Conclusion

In conclusion, we observed excellent outcome of patients with MNC-MC because of the absence of significant GvHD and no increased relapse risks whereas Gran-MC was associated with inferior outcome because of higher relapse risks in agreement with the hypothesis that recipient type myelopoiesis may reflect persistent malignancy. Outcome of FC patients was intermediate. MC proved to be long-lasting in a significant minority of patients, mainly with T-cell depletion, remaining stable for decades in some.

## Figures and Tables

**Table 1:** Distribution of chimerism status based on main transplant features.

	FC	MNC-MC	Gran-MC	Total	*p* value
	(*n* = 89)	(*n* = 36)	(*n* = 30)	(*n* = 155)	
Gender					
Female (F)	29 (0.19)	16 (0.1)	17 (0.11)	62 (0.4)	0.055
Male (M)	60 (0.39)	20 (0.13)	13 (0.08)	93 (0.6)	
Disease					
ALL	16 (0.1)	6 (0.04)	3 (0.02)	25 (0.16)	0.739
AML	27 (0.17)	14 (0.09)	12 (0.08)	53 (0.34)	
CLL	3 (0.02)	0	0	3 (0.02)	
CML	17 (0.11)	8 (0.05)	7 (0.04)	32 (0.21)	
Lymphoma	10 (0.07)	5 (0.03)	1 (0.01)	16 (0.1)	
MDS	8 (0.05)	2 (0.01)	2 (0.01)	12 (0.08)	
MPN	2 (0.01)	0	1 (0.01)	3 (0.02)	
Myeloma	6 (0.04)	1 (0.01)	4 (0.03)	11 (0.08)	
Conditioning					
Standard	77 (0.5)	33 (0.21)	25 (0.16)	135 (0.87)	0.585
Reduced	12 (0.08)	3 (0.02)	5 (0.03)	20 (0.13)	
Donor type					
Identical sibling	62 (0.4)	29 (0.19)	22 (0.14)	113 (0.73)	0.263
Unrelated	21 (0.14)	7 (0.04)	8 (0.05)	36 (0.23)	
Mismatched related	6 (0.04)	0	0	6 (0.04)	
Donor/recipient sex match					
F → F	17 (0.11)	10 (0.07)	2 (0.01)	29 (0.19)	**0.001**
F → M	29 (0.19)	5 (0.03)	6 (0.04)	40 (0.26)	
M → F	12 (0.08)	6 (0.04)	15 (0.1)	33 (0.21)	
M → M	31 (0.2)	15 (0.1)	7 (0.04)	53 (0.34)	
T-cell depletion					
None	19 (0.12)	1 (0.01)	0	20 (0.13)	**<0.001**
T-cell depletion without add-back	48 (0.31)	16 (0.1)	12 (0.08)	76 (0.49)	
T-cell depletion with add-back	22 (0.14)	19 (0.12)	18 (0.12)	59 (0.38)	
Stem cells source					
Bone marrow	26 (0.17)	15 (0.1)	15 (0.1)	56 (0.36)	0.09
Peripheral blood	63 (0.41)	21 (0.14)	15 (0.1)	99 (0.64)	
aGvHD					
Grades 0-1	60 (0.39)	31 (0.2)	25 (0.16)	116 (0.75)	**0.045**
Grades 2–4	29 (0.19)	5 (0.03)	5 (0.03)	39 (0.25)	
cGvHD					
None	52 (0.34)	32 (0.21)	24 (0.16)	108 (0.7)	**0.009**
Limited	21 (0.14)	3 (0.02)	3 (0.02)	27 (0.17)	
Extensive	16 (0.1)	1 (0.01)	3 (0.02)	20 (0.13)	
DLI					
0	68 (0.44)	29 (0.19)	20 (0.13)	117 (0.76)	0.406
≥1	21 (0.14)	7 (0.04)	10 (0.07)	38 (0.24)	
